# Positional changes of the third molar in orthodontically treated patients

**Published:** 2013-06-25

**Authors:** AM Mihai, IR Lulache, R Grigore, AS Sanabil, S Boiangiu, E Ionescu

**Affiliations:** Department of Orthodontics and Dento-Facial Orthopedics, “Carol Davila" University of Medicine and Pharmacy, Bucharest

**Keywords:** Third molar, premolar, extraction

## Abstract

Objective and Rationale. Over the years, the effects of the third molars eruption on the dental arches have been studied extensively. Still, literature provides less data regarding the effects of the orthodontic treatment on the third molars position. The aim of our study was to assess the positional changes of the third molars relative to the occlusal plane and to the second molar long axis, changes occurred during orthodontic treatment performed with or without premolar extractions.

Method. This study included 20 orthodontic treated patients: 10 of them with premolar extractions and 10 without premolar extractions. The pretreatment and post treatment panoramic radiographs were analyzed, and the angles between the third molar long axis and the occlusal plane and between the long axis of the third molar and the long axis of the second molar were measured.

Results. Changes in third molar position, from pretreatment to post treatment, for the two groups of patients were evaluated by using the Student’s t-test. The results of the statistical analysis revealed an improvement in third molars position, the best results were seen in the lower third molars, in the group of patients treated with premolar extractions.

## Introduction

The wisdom teeth (M3) hold a unique position in the dentofacial complex because of their formation, development and evolution. Generally, they begin their formation at about 8-9 years of age (with a degree of variation ranging from 5-14 years) [**1,4**] and emerge into the oral cavity around the age of 18-24 years, presenting a considerable variation as in the case of formation [**1,4-6**]. 

 In orthodontics, it is well known that special attention should be given to third molars. Regarding these teeth, the main issues that need to be considered are related to the possibility of their eruption into the oral cavity. It also raises the question if wisdom teeth will cause the crowding of the anterior teeth and whether the extraction of other teeth could prevent this crowding, influencing subsequent favorably eruption of wisdom teeth [**7**]. 

 Many existing studies demonstrate the constant concern of the dentistry, in general, and orthodontics, in particular, for the third molars. In general, these studies have sought to investigate the effects of the eruption of wisdom teeth on the dental arches. Literature provides less data regarding the effects of orthodontic treatment performed with or without premolar extractions, on third molars position. In some of these studies, premolar extractions treatment was associated with a high mesial drift of the third molar while non-extraction treatment was associated with a significant increase in the frequency of the third molar impaction; there are also authors who have not found significant differences in patients treated with or without premolar extractions [**1,6,8**]. 

 Ironically called the "wisdom tooth", the third molar is considered a causative factor of many complications, although its role in such complications was not fully established until now [**1-7**]. 

 One of its pathological implications is the phenomenon of impaction. Frequency of impaction of this tooth shows a high degree of variability. Depending on the studied population, it is between 9.5 and 25%, considering that the lack of space available for its eruption is the main cause of this dental anomaly [**1,5,6,9-11**]. 

 Another explanation of the wisdom tooth impaction can be given by the disturbances that occur during the formation of the tooth. It is known that, during development, the wisdom teeth permanently change their inclination [**12**] and undergo important pre-eruptive rotational movements [**13**]. These rotational movements occur when the third molar bud gets close to the second molar (M2). Thus, at the beginning of calcification, the third molars buds are angulated mesially in the mandible and distally in the maxillary (Sicher, 1965). Richardson [**10**] found that between 10 and 15 years, there is an average displacement of 11.2⁰ of the mandibular third molar with respect to the mandibular plane [**7**]. This indicates a tendency for the tooth to become more upright, with the angle of the mandibular third molar to the occlusal plane tending to decrease. In case the rotational movements fail to occur, we may face the phenomenon of impaction [**7,13**]. 

 There are authors who consider that these uprighting rotational movements may be stimulated by extraction of teeth that are placed mesially to the third molar [**7,13**]. 

 For these reasons, orthodontists should take into consideration the link existing between the third molars and the rest of the dentition. 

Considering these aspects, the aim of our study was to assess the positional changes of the third molars relative to the occlusal plane and to the second molar long axis, changes occurred during orthodontic treatment performed with or without premolar extractions.

## Materials and methods

 For this study, a sample of 20 patients (12 girls and 8 boys) who undergone fixed orthodontic treatment at the Department of Orthodontics and Dento-Facial Orthopedics of “Carol Davila" University in Bucharest was selected. The sample of patients was divided into two sub-groups: a group of 10 patients who have received orthodontic treatment without premolar extractions and a second group of 10 patients treated with premolar extractions. The inclusion criteria in the study group were the following:

 - Patients with Class I malocclusion at the start of treatment;

 - Existence of pretreatment (T1) and post treatment (T2) panoramic radiographs;

 - The crowns of the third molar formed at the beginning of the orthodontic treatment;

 - Second premolars (Pm2) fully erupted into the mouth;

 - Patients treated with maxillary and mandibular straight-wire appliances, without additional anchorage preparation and without the use of special systems for mesial or distal migration.

 The pretreatment (T1) and post treatment (T2) panoramic radiographs were evaluated by using the technique of tracing the images of the teeth on the matte acetate paper and the following angular measurements were made:

### 1. The anterior angle between the long axis of the third molar and the occlusal plane (∡M3-OP)

 The long axes of the third molar buds were considered the line bisecting the angle formed by the tangent to the mesial and the distal outlines of the molar. The line connecting the mesiobuccal cusps of the first molar with the buccal cusps of the second premolar defined the occlusal plane;

### 2. The anterior angle formed between the long axis of the third molar and the long axis of the second molar (∡M3-M2)

 For the third molar long axis, we used the representation described above, in the case of the M3-OP angle. The second molar long axis was defined by the projection of the midpoint of the root bifurcation on the line connecting the two buccal cusps.

 In order to evaluate the changes that occurred during the orthodontic treatment, we calculated the differences between the post treatment and the pretreatment values (T2-T1) for each measurement. The changes in the third molar angulation, relative to the occlusal plan and relative to the second molar, changes that occurred during the orthodontic treatment, were evaluated, for each group of patients, by using the Student’s t-test.

**Fig. 1:**
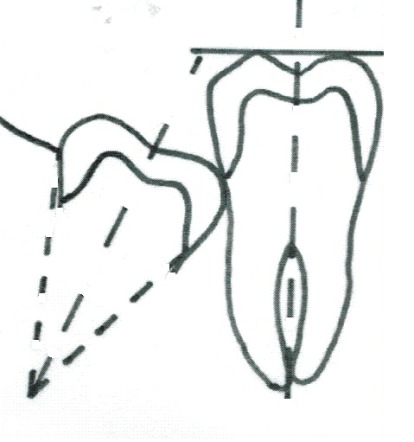
Third molar long axis determination

**Fig. 2:**
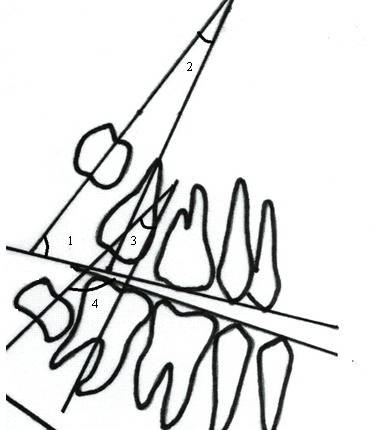
Angular variables determination (1) the anterior angle between the long axis of the maxillary third molar and the occlusal plane (M3-PO); (2) the angle between the long axes of the maxillary second and third molars (M3-M2); (3) the angle between the long axes of the mandibular second and third molars (M3-M2); (4) the anterior angle between the long axis of the mandibular third molar and occlusal plane (M3-PO)

## Results

 In order to appreciate the changes of the third molars position, which occurred during orthodontic treatment, we considered the fact that, in the upper jaw, an increase of the M3-OP angle and a decrease of the M3-M2 angle indicate a favorable change in the maxillary third molar angulation, while a decrease of the M3-PO angle and an increase of the M3-M2 angle shows an unfavorable change of the third molar position. In the lower jaw, the reduction of the angles M3-OP and M3-M2 are considered signs of favorable development of the third molar position, indicating a straightening movement of the tooth, with increased subsequent chances of eruption into the mouth.

 The results of the Student’s t-test regarding the changes in the third upper and lower molar position, that occurred during orthodontic treatment, are shown in Table 1 and Table 2

**Table 1:** Changes in mandibular third molar angulation relative to the occlusal plane and to the second molar long axis (median values).

		Extractions (median values)			Mandible Nonextractions (median values)			Differences (median values)	
	Before Treatment	After Treatment	P	Before Treatment	After Treatment	P	Extractions	Nonextractions	P
M3-OP	123,7	113,75	*	126,75	118,4	*	-9,95	-8,35	ns
M3-M2	26,65	21,4	ns	18	17,85	ns	-5,25	-0,15	ns
* p<0.05 ns – not significant									

 Based on the observation that, at the start of the treatment, the angular measurements values showed no significant differences between the two groups, Table 1 shows the statistical analysis results regarding the changes of the mandibular third molar position relative to the occlusal plane and to the second molar long axis. Examining the data presented in this table, a decrease of the angle between the long axis of the lower third molar and the occlusal plane can be noticed. The results showed statistically significant values for both groups of patients. However, the decrease of the angle between the third molar and the occlusal plane is more important for the group of patients treated with premolar extractions.

Also, the results of this study showed that during the orthodontic treatment, a decrease of the angle formed by the long axis of the mandibular third molars relative to the second molar occurred, both in patients treated with and without premolar extractions. The decrease of this angle was more important in the extraction group, but the results were not statistically significant.

 Therefore, comparing the group of patients treated with premolar extraction with the group of patients who were treated without premolar extractions, the analysis for the lower jaw showed statistically insignificant differences in terms of the angle formed by the long axis of the mandibular third molar relative to the occlusion plan and to the second molar long axis.

**Table 2:** Changes in the maxillary third molar angulation relative to the occlusal plane and to the second molar long axis (median values).

		Extractions (median values)			Maxilla Nonextractions (median values)			Differences (median values)	
	Before Treatment	After Treatment	P	Before Treatment	After Treatment	P	Extractions	Nonextractions	P
M3-OP	72,2	76,05	ns	70,3	72,5	ns	3,85	2,2	ns
M3-M2	11,35	10,25	ns	11,5	17,75	ns	-1,1	6,25	ns
* p<0.05 ns – not significant									

 Table 2 lists the descriptive statistics for changes in the maxillary third molars position, changes occurred during the orthodontic treatment. Analyzing the values obtained, it was observed that, during the treatment, the angle formed by the third molar relative to the occlusal plan, increased in both groups, signifying an uprighting of the third molar. However, the results show that the uprighting of the wisdom tooth long axis is more important in patients treated with premolar extractions.

 The angle between the maxillary third and the second molar has decreased, the third molar axis has uprighted, but only in patients treated with premolar extractions. In the group of patients treated without extractions, the results showed increased values of this angle, pointing an unfavorable change in the wisdom tooth angulation.

 In both groups of patients, the changes in maxillary third molars angulation resulting from orthodontic treatment showed no statistically significant values.

 Comparing the upper and the lower jaw, it can be concluded that after the orthodontic treatment, the third molars showed an improvement in angulation, and this is more evident in the group of patients treated with premolar extractions especially for the mandibular third molars.

## Discussion

 By gathering and putting together the observations of this study that assessed the changes in the third molar the angulation relative to the occlusal plane and to the second molar long axis, changes occurred during orthodontic treatment performed with or without premolar extractions, we may affirm that, in most cases after treatment, the third molar angulation improved. In our study, the best results were seen in the lower third molars, in the group of patients treated with premolar extractions.

 Regarding these coordinates, our findings coincide with the other studies in literature. Thus, authors such as Richardson [**2**], Kim [**4**] and Jain [**7**] found that the premolar extraction increases the available space in the molar area, having as a result an improvement of the wisdom tooth position. On the other hand, Haavikko [**14**] states that premolar extractions accelerates, but does not favor the eruption of the wisdom teeth. 

 Since the third molar position improved, whether or not premolars were extracted, the results of our study do not reveal other predictive factors for the wisdom teeth eruption. 

 The type of mechanics used during orthodontic treatment may have had an influence on the third molar angulation. All the patients included in our study had dentally and skeletally Class I malocclusion, and, during the treatment, no additional anchorage devices were used; if the patients had presented Class II dental reports, in cases treated with premolar extractions, lower molars would have been protracted in order to achieve the class I molar report and the third molar axis could pose a more important degree of uprighting. Also, if a Class II malocclusion had been treated by an upper molar distalization, after the treatment, the third molar angulation would have changed in a negative way.

 Following this study, we cannot affirm with certainty that premolar extractions treatment would ensure subsequent eruption of the wisdom teeth, and this because other factors may influence the inclination and the eruption of these teeth.

 Thus, the post-orthodontic growth and the initial angulation of the wisdom teeth could facilitate their subsequent eruption. Equally, it should be kept in mind that the changes in the occlusal plane, which may occur during the orthodontic treatment, may cause misinterpretation of the third molar angle calculations.

 Also, it seems that the initial angulation of the third molars may influence their subsequent eruption. Richardson [**2**], following his studies, found that mandibular third molars with a small degree of angulation relative to the occlusal plane erupted earlier than those with steeper angulations.

 Mandibular growth rate and third molars mineralization can be considered as other factors that contribute to the third molars eruption, but their exact role is still uncertain [**6**].

 Therefore, when designing the treatment plan, it is prudent for the orthodontist to inform their patients that premolar extractions does not guarantee the third molar eruption and that they will have enough space for a good alignment into the dental arch. Also in non extraction cases, it must be taken into account the fact that, although the position of third molar can be improved, the eruption is still uncertain, which could cause a case without extraction to become in the future a case treated also by extraction, but wisdom tooth extraction this time.

## References

[1] Tarazona  B, Paredes  V (2010). Influence of first and second premolar extraction or non-extraction treatments on mandibular third molar angulation and position. A comparative study. Medicina Oral Patologia Oral y Cirugia Bucal.

[2] Richardson  ME (1974). Some aspects of lower third molar eruption. The Angle Orthodontis.

[3] 3. Kaplan  RG (1975). Some factors related to mandibular third molar impaction. The Angle Orthodontist.

[4] Kim  TW, Artun  J (2003). Prevalence of third molar impaction in orthodontic patients treated nonextraction and with extraction of 4 premolars. American Journal of Orthodontics and Dentofacial Orthopedics.

[5] Artun  J, Thalib  L (2005). Third molar angulation during and after treatment of adolescent orthodontic patients. European Journal of Orthodontics.

[6] Staggers  JA, Germane  N (1992). A comparison of the effects of first premolar extractions on third molar angulation. The Angle Orthodontist.

[7] Jain  S, Valiathan  A (2009). Influence of First Premolar Extraction on Mandibular Third Molar Angulation. The Angle Orthodontist.

[8] Richardson  ME (1989). The effect of mandibular first premolar extraction on third molar space. The Angle Orthodontist.

[9] Altonen  M, Haavikko  K (1977). Developmental position of lower third molar in relation to gonial angle and lower second molar. The Angle Orthodontist.

[10] Richardson  ME (1970). The early developmental position of the lower third molar relative to certain jaw dimensions. The Angle Orthodontist.

[11] Richardson  ME (1973). Development of the lower third molar from 10 to 15 years. The Angle Orthodontist.

[12] Richardson  M (1978). Pre-eruptive movements of the mandibular third molar. The Angle Orthodontist.

[13] Silling  G (1973). Development and eruption of the mandibular third molar and its response to orthodontic therapy. The Angle Orthodontist.

[14] Haavikko  K, Altonen  M (1978). Predicting angulational development and eruption of the lower third molar. The Angle Orthodontist.

